# Profiling of proteins secreted in the bovine oviduct reveals diverse functions of this luminal microenvironment

**DOI:** 10.1371/journal.pone.0188105

**Published:** 2017-11-20

**Authors:** Viju Vijayan Pillai, Darren M. Weber, Brett S. Phinney, Vimal Selvaraj

**Affiliations:** 1 Department of Animal Science, College of Agriculture and Life Sciences, Cornell University, Ithaca, New York, United States of America; 2 Genome Center, Proteomics Core Facility, University of California, Davis, Davis, California, United States of America; Friedrich-Loeffler-Institute, GERMANY

## Abstract

The oviductal microenvironment is a site for key events that involve gamete maturation, fertilization and early embryo development. Secretions into the oviductal lumen by either the lining epithelium or by transudation of plasma constituents are known to contain elements conducive for reproductive success. Although previous studies have identified some of these factors involved in reproduction, knowledge of secreted proteins in the oviductal fluid remains rudimentary with limited definition of function even in extensively studied species like cattle. In this study, we used a shotgun proteomics approach followed by bioinformatics sequence prediction to identify secreted proteins present in the bovine oviductal fluid (*ex vivo*) and secretions from the bovine oviductal epithelial cells (*in vitro*). From a total of 2087 proteins identified, 266 proteins could be classified as secreted, 109 (41%) of which were common for both *in vivo* and *in vitro* conditions. Pathway analysis indicated different classes of proteins that included growth factors, metabolic regulators, immune modulators, enzymes, and extracellular matrix components. Functional analysis revealed mechanisms in the oviductal lumen linked to immune homeostasis, gamete maturation, fertilization and early embryo development. These results point to several novel components that work together with known elements mediating functional homeostasis, and highlight the diversity of machinery associated with oviductal physiology and early events in cattle fertility.

## Introduction

The oviductal microenvironment is a site for key events that involve gamete maturation, fertilization and early embryo development, processes that ultimately determine reproductive success. The oviductal epithelium has long been known to secrete specific proteins and metabolic elements, which in addition to components derived from blood plasma forms the oviductal fluid [1, 2]. In recent years, there has been accumulating evidence that several of these protein components might contribute to developmental events that occur in this microenvironment [3, 4]. In support, supplementing oviductal fluid components during *in vitro* fertilization (IVF) has demonstrated improved fertilization success and development rates [5–7]; co-culture with bovine oviductal epithelial cells (OECs) during IVF has indicated positive effects on early development of embryos [8–12]. These effects have been particularly linked to regulation of metabolic pathways [10, 13, 14], and in some cases epigenetic modulation of the developing embryo [11, 15]. Nevertheless, a comprehensive evaluation of secreted proteins in the oviductal fluid remains to be conducted, and data exist only from targeted studies with limited definition of function even in extensively studied species like cattle [16, 17]

Early embryonic loss is a major basis for reduced fertility in cattle [18]. Following fertilization, the embryo resides in the oviductal microenvironment for the first 3–4 days of development, during which sequential cleavage leading up to the 16-cell stage occurs before the embryo enters the uterus [19]. Efforts to study bovine oviductal fluid components started in the late 1950s [20], with initial focus on total protein content and free amino acid levels [21–23], and concentrations of metabolic components [21, 24]. Subsequent studies examining specific proteins in bovine oviductal fluid have largely taken topical or focused approaches, for example, visualizing proteins that associate with gametes [25–27], immuno-identification of glycoproteins synthesized at estrus [28, 29], insulin-like growth factors and binding proteins [30]. Proteomic profiling of components in the oviductal fluid and uterine fluid have been performed in other farm animal species like pigs [31, 32], and this has led to improvement of *in vitro* embryo production methods [33]. However, potential proteins that could be present in the bovine oviductal fluid have only been extrapolated from gene expression studies on the oviductal epithelium [34–36].

Knowledge of the bovine oviductal microenvironment and its effect on physiology of early embryo development would be important for improving *in vitro* embryo production methods and perhaps identifying unique bovine pluripotency mediators. In the present investigation, we use a shotgun proteomics approach to identify and compare secreted proteins in the bovine oviductal fluid, and secretions from OECs in culture with and without stimulation. Our results reveal several novel components that highlight the diversity of functions associated with the oviductal microenvironment. These findings represent the first step towards improved understanding of factors that could influence early events in cattle fertility.

## Materials and methods

### Animals and reagents

Samples from Holstein cows (*Bos taurus*) were collected from the slaughterhouse (Cargill^®^, Wyalusing, PA). Healthy reproductive tracts in both follicular and luteal phases of the estrous cycle were included in this study. All reagents were purchased from Sigma-Aldrich (St Louis, MO), unless otherwise noted.

### Collection of oviductal fluid

Reproductive tracts were removed immediately after slaughter and both oviducts were isolated for collection at random stages of the estrous cycle. Using a fire-polished glass Pasteur pipette, fluid from the ampulla and isthmus of 28 cows (10–30 μl per animal) was collected by gentle aspiration. Fluid collected were combined and centrifuged at 2000 x g for 5 minutes, supernatant then removed and filtered using a low protein binding 0.2 μm polyethersulfone syringe filter to remove any cells or debris. Samples were snap-frozen in liquid nitrogen and held at -80°C until further processing. The sample collection procedure was completed within 30 minutes after slaughter of the animal.

### Culture and characterization of OECs

**Fig 1:**
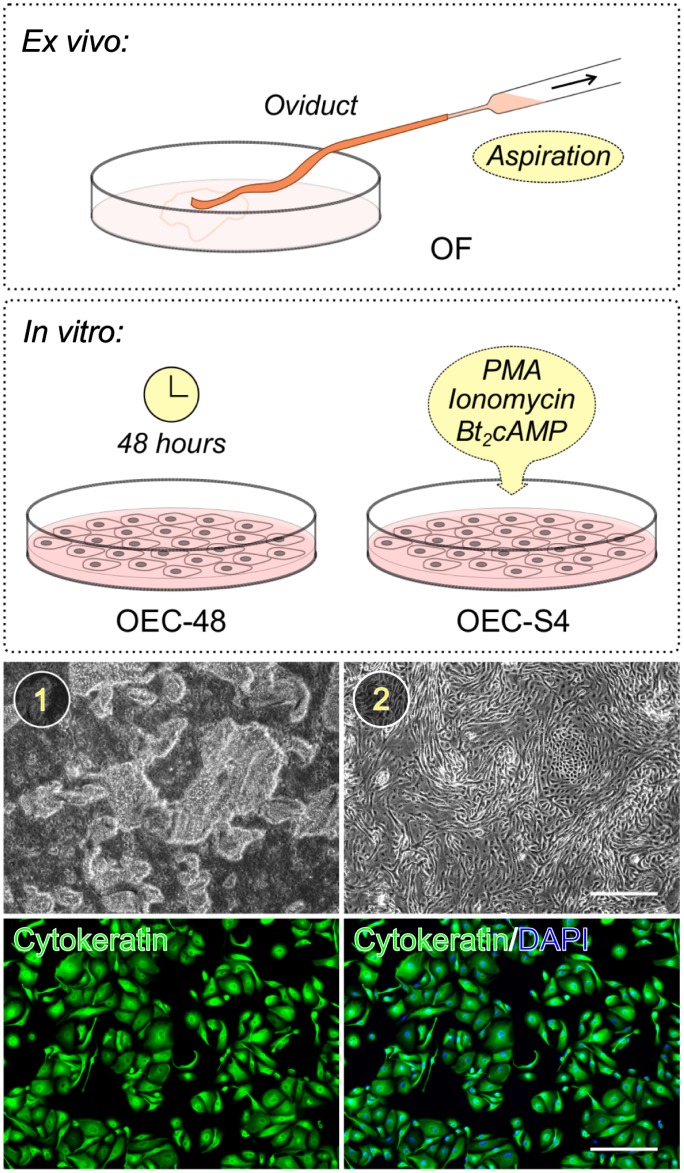
Collection of oviductal cell secretions. Oviducts were dissected and removed from the mesosalphinx and fluid was collected either by direct aspiration (*ex vivo*) or after the culture of the oviductal epithelium (*in vitro*). Media used for culturing oviductal epithelial cells (OECs) were collected after passive conditioning for 48 hours or after a 4-hour stimulation with phorbol myristate acetate (PMA), ionomycin and dibutyryl cyclic adenosine monophosphate (Bt_2_cAMP). (1) Extruded oviductal mucosa containing intact epithelial sheets. (2) Representative image showing attachment and growth of the oviductal epithelial cells after 2–3 days in culture (Scale bar 300 μm). Immunohistochemistry for cytokeratin as a marker for OECs showed that the cultures established by this method were of high purity (Scale bar 200 μm).

Intact oviducts together with the tip of the uterine horn and the ovary were dissected immediately after slaughter and transported on ice in Dulbecco’s modified eagle medium (DMEM) containing 10 mM 2-[4-(2-hydroxyethyl)piperazin-1-yl]ethanesulfonic acid (HEPES) and penicillin-streptomycin supplement. Chilled oviducts were dissected from the surrounding connective tissue and washed several times using phosphate buffered saline (PBS). The region of ampulla and isthmus were trimmed and retained in petridishes with M199 medium containing penicillin streptomycin. The oviductal mucosa that contains the epithelial layer was gently extruded by mechanical pressure using atraumatic forceps and collected in a separate tissue culture dish containing the same medium in pairs (S1 Movie). The cell aggregates were then gently dispersed using a fire-polished Pasteur pipette and centrifuged at 500 x g for 5 minutes in a swinging bucket centrifuge. The cell clusters were suspended in complete culture medium M199 containing 10% fetal bovine serum, 1% non-essential amino acids supplement and 1% penicillin-streptomycin and plated in tissue culture dishes and incubated at 37°C under an atmosphere of 5% CO_2_ to allow attachment and proliferation. Cell morphology and growth was assessed visually, and subsequently OECs were evaluated for expression of the epithelial marker cytokeratin (Fig 1).

### Immunocytochemistry

Primary bovine OECs were grown on coverslips coated with 0.2% gelatin and fixed with 4% formaldehyde. Cells were then permeabilized with 0.1% Triton X-100 in PBS for 1 minute and blocked using 5% normal goat serum for 30 minutes. Coverslips were subsequently incubated with a mouse monoclonal anti-cytokeratin antibody (1:200 dilution; Cell Signaling Technology) for 1 hour. Coverslips were then washed three times using PBS and incubated with Alexa Fluor conjugated anti-mouse Fab’ fragments for 30 minutes, washed again with PBS, counterstained/mounted with 4’,6-diamidino-2-phenylindole (DAPI) containing Prolong Gold reagent (Life Technologies, Carlsbad, CA). Images were acquired using an inverted microscope (DMI 3000, Leica) using a cooled monochromatic camera (DFC365FX, Leica).

### Collection of OEC conditioned media

Confluent OEC mixed cultures from at least 10 different animals were used for generating two types of OEC conditioned media mainly from apical secretions. First, adherent cells were washed with two repeated changes of PBS followed by two repeated changes of serum free M199 medium. For the 48-hour collection period (OEC-48), plates were returned to the incubator and kept undisturbed for that duration. For the stimulated secretions (OEC-S4), fresh serum free M199 medium supplemented with 5 ng/ml phorbol myristate acetate (PMA), 500 ng/ml of ionomycin and 0.5 mM of dibutyryl cyclic adenosine monophosphate (Bt_2_cAMP) was added to the cells and incubated for 4 hours. Use of these secretagogues were to enhance protein secretions mimicking cell activation signals that induce protein kinase A, protein kinase C, and increase intracellular Ca^2+^ levels. At the end of the incubation period, media were collected from the dishes, centrifuged at 500 x g for 5 minutes, filtered using a 0.2 μm PES syringe filter, snap frozen in liquid nitrogen and stored at -80°C until further processing.

### Sample preparation and digestion

Protein concentrations were determined using BCA assay kit following manufacturer directions. Samples were then precipitated using the ProteoExtract Protein Precipitation Kit (CalBiochem). Resulting protein pellet was solubilized in 6 M urea in 50mM ammonium bicarbonate. Dithiothreitol (DTT) was added to a final concentration of 5 mM and samples were incubated for 30 min at 37°C. Subsequently, 20 mM iodoacetamide (IAA) was added to a final concentration of 15 mM and incubated for 30 min at room temperature, followed by the addition of 20 μL DTT to quench the IAA reaction. Lys-C/trypsin (Promega) was used at a 1:25 ratio (enzyme:protein) and incubated at 37°C for four hours. Samples were then diluted to <1 M urea by the addition of 50 mM ammonium bicarbonate and digested overnight at 37°C. The following day, samples were desalted using C18 Macro Spin columns (Nest Group) and dried down by vacuum centrifugation.

### LC-MS/MS analysis

LC separation was done on a Proxeon Easy-nLC II HPLC (Thermo Scientific) with a Proxeon nanospray source. The digested peptides were reconstituted in 2% acetonitrile/0.1% trifluoroacetic acid and 10 μl of each sample was loaded onto a 100 μm x 25 mm Magic C18 100Å 5U reverse phase trap where they were desalted online before being separated on a 75 μm x 150 mm Magic C18 200Å 3U reverse phase column. Peptides were eluted using a gradient of 0.1% formic acid and 100% acetonitrile with a flow rate of 300 nL/min. A 120-min gradient was run with 5% to 35% acetonitrile over 100 min, 35% to 80% acetonitrile over 10 min, 80% acetonitrile for 2 min, 80% to 5% acetonitrile over 5 min, and finally held at 5% acetonitrile for 5 min.

Mass spectra was collected on an Orbitrap Q Exactive mass spectrometer (Thermo Fisher Scientific) in a data-dependent mode with one MS precursor scan followed by 15 MS/MS scans. A dynamic exclusion of 5 sec was used. MS spectra were acquired with a resolution of 70,000 and a target of 1 × 10^6^ ions or a maximum injection time of 20 msec. MS/MS spectra were acquired with a resolution of 17,500 and a target of 5 × 10^4^ ions or a maximum injection time of 250 msec. Peptide fragmentation was performed using higher-energy collision dissociation (HCD) with a normalized collision energy (NCE) value of 27. Unassigned charge states as well as +1 and ions > +5 were excluded from MS/MS fragmentation.

### Database searching

Tandem mass spectra were extracted by Proteome Discoverer v1.2. Charge state deconvolution and deisotoping were not performed. All MS/MS samples were analyzed using X! Tandem (The GPM, www.thegpm.org; version CYCLONE 2013.02.01.1). X! Tandem was set up to search the Uniprot bovine proteome (23,942 entries) and 116 common laboratory contaminants (www.thegpm.org/crap) with an equal number of reverse decoy sequences assuming the digestion enzyme trypsin. X! Tandem was searched with a fragment ion mass tolerance of 20 PPM and a parent ion tolerance of 20 PPM. Carbamidomethyl of cysteine was specified in X! Tandem as a fixed modification. Glu->pyro-Glu of the n-terminus, ammonia-loss of the n-terminus, gln->pyro-Glu of the n-terminus, deamidated of asparagine and glutamine, oxidation of methionine and tryptophan, dioxidation of methionine and tryptophan and acetyl of the n-terminus were specified in X! Tandem as variable modifications.

### Criteria for protein identification

Scaffold (version 4.2.0, Proteome Software Inc., Portland, OR) was used to validate MS/MS based peptide and protein identifications. Peptide identifications were accepted if they could be established at greater than 79.0% probability by the Scaffold Local FDR algorithm. Protein identifications were accepted if they could be established at greater than 95.0% probability to achieve an FDR less than 5.0% and contained at least 2 unique peptides. This resulted in a spectra decoy FDR of 0.35% and a protein decoy FDR of 4.9%. Protein probabilities were assigned by the Protein Prophet algorithm [37]. Proteins that contained similar peptides and could not be differentiated based on MS/MS analysis alone were grouped to satisfy the principles of parsimony. Proteins sharing significant peptide evidence were grouped into clusters.

### Analysis of identified proteins

To predict proteins secreted via the classical secretory pathway in the resulting dataset, we used SignalP v4.1 (http://www.cbs.dtu.dk/services/SignalP/) [38] to identify N-terminal sequence motifs directing proteins to the secretory pathway, in tandem we used TargetP v1.01 (www.cbs.dtu.dk/services/TargetP) [39] to refine this dataset by removing proteins destined for the mitochondria [40]. This predicted dataset was further refined using Phobius (http://phobius.sbc.su.se/) [41] to remove proteins that contained transmembrane regions. In this overall analysis, proteins were considered secreted if they contained an N-terminal secretory sequence, did not traffic to the mitochondria, and lacked transmembrane regions. In parallel, to predict proteins secreted via the non-classical secretory pathway in the same dataset, we used SecretomeP v2.0 (http://www.cbs.dtu.dk/services/SecretomeP/) [42] for feature-based analysis and identification of secreted proteins that do not contain an N-terminal signal sequence motif. Results from SecretomeP were further filtered using Phobius as described above. In this pipeline, glycosylphosphatidylinositol anchored surface proteins would also be identified as secreted. The resulting protein lists were classified by using gene ontology (GO) terms using PANTHER (protein analysis through evolutionary relationships tool [43]. For integrated functional evaluation, the proteins identified were also analyzed using Ingenuity^®^ pathway analysis (IPA) to model and interpret biological significance of identified components. Common candidates in the proteomics dataset and from reanalysis of two published transcriptomics datasets from *Bos taurus* (NCBI GEO: GSE74612 [44]), and *Bos indicus* (GEO GSE65681 [36]), were identified and visualized as Circos plots [45], together with the classification based on GO terms.

### Data availability

Raw data, mzML and Scaffold results are available from the MassIVE proteomics repository (MSV000081192) and Proteome Exchange (PXD006794). Complete protein lists are provided in supporting information (S1 Dataset).

## Results and discussion

### Secreted proteins in the bovine oviductal fluid

**Fig 2:**
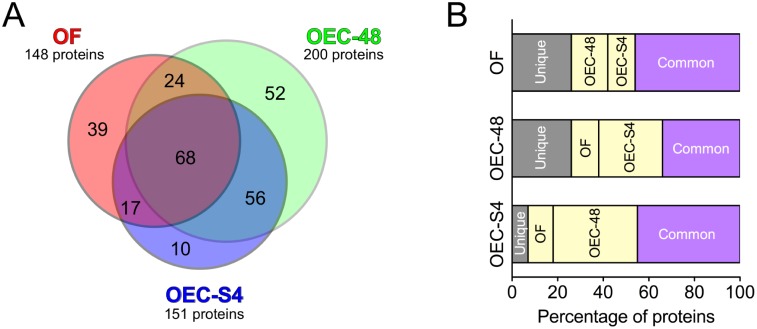
Comparison of proteins identified under different collection methods for oviductal cell secretion conditions. A total of 266 secreted proteins were identified. (A) Distribution of proteins that were unique or common between the three different groups: oviductal fluid (OF) and conditioned media obtained from oviductal epithelial cell cultures without (OEC-48) or after stimulation (OEC-S4). A subset of 68 proteins was common for the three groups. OEC-S4 had the least number of unique proteins that were not represented in OF or OEC-48. (B) Distribution of the percentage of unique and common proteins within each of the three collection groups.

Experimental groups in this study enabled the identification of proteins that were secreted under *ex vivo* (OF) and *in vitro* (OEC-48 and OEC-S4) conditions (Fig 1). Cytokeratin expression evaluated in the *in vitro* cultures showed that OECs were of high purity without any contaminating fibroblasts (Fig 1). Protein mass spectrometric analysis identified a total of 2087 proteins combined for the three groups: 1289 proteins in OF, 1148 proteins in OEC-48, and 1391 proteins in OEC-S4. Within this combined list that would include proteins present within exosomes or released from damaged cells, 266 proteins passed the SignalP, TargetP, SecretomeP and Phobius filters indicating the putative number of secreted proteins identified: 148 proteins in OF, 200 proteins in OEC-48, and 151 proteins in OEC-S4. Of these, 68 proteins (26%) were common for all three groups (S1 Table), 109 proteins (41%) were common between *in vivo* and *in vitro* conditions, and 165 proteins (62%) were common for at least two of the groups (Fig 2A and 2B, S1 Dataset). We did not detect immunoglobulins in any of the samples demonstrating that our sampling method was without plasma/serum contamination.

**Fig 3:**
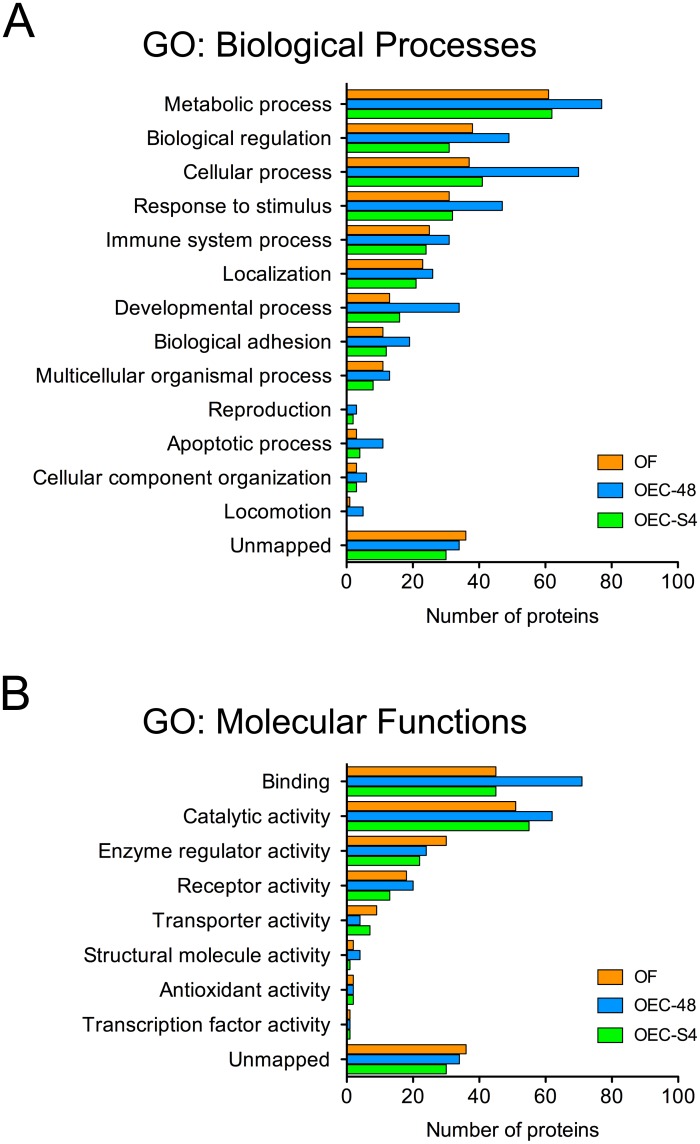
Gene ontology (GO) classification for all proteins identified in oviductal cell secretions. Distribution of GO terms describing (A) biological processes, and (B) molecular functions for the number of identified secreted proteins.

**Fig 4:**
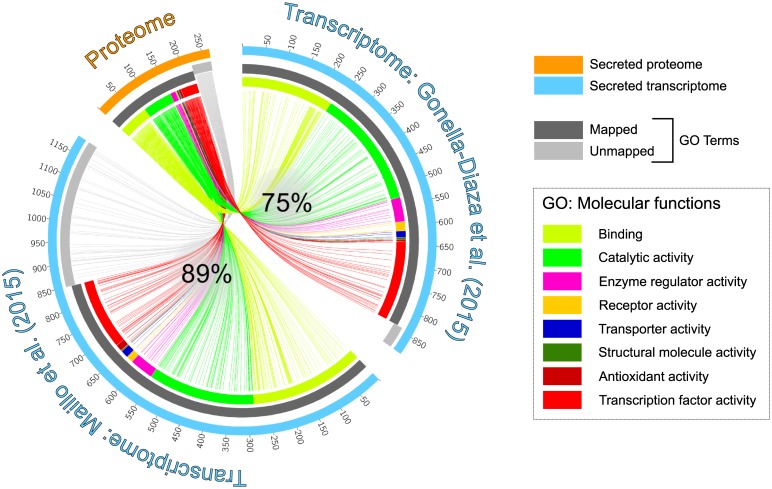
Comparison of oviductal cell secreted proteins to transcriptome of the bovine oviduct from two published datasets. Of the 266 secreted proteins identified in oviductal cells, 236 (89%) and 200 (75%) of the proteins were detected as transcripts in the oviduct by Maillo *et al*. [35], and the Gonella-Diaza *et al*. [36] respectively. This comparison indicates that almost 90% of the proteins identified in this study are synthesized by the oviductal epithelium, with only 30 (11%) of proteins putatively derived by plasma protein transudation.

General categories of function for proteins identified in the oviductal secretome based on biological process or molecular function (Fig 3) were aligned to the major physiological processes that occur in the oviductal lumen. When the list of secreted proteins identified were compared to oviduct transcriptomics data from two different studies, we identified 236 (89%) of the proteins identified also represented as transcripts in Maillo *et al*. [35], and 200 (75%) in Gonella-Diaza *et al*. [36] (Fig 4). This also highlighted an important finding that most of the proteins detected were synthesized and secreted by the oviductal epithelium, and that there was little contribution of plasma protein components to the oviductal fluid (S2 Table). This is in contrast to a previous observation made in rabbits in which immunoglobulins and albumin were identified as major components [46]. We can only speculate the reason underlying this distinction, and one possibility is the species differences that are known to impact events that occur in the oviduct.

The list of proteins and the experimental group in which they were detected are presented based on broad categorizations as, growth factors and cytokines integral to this functional niche (Table 1), homeostasis maintained by protease and protease inhibitors (Table 2), other enzymes involved in a variety of functions (Table 3), other proteins associated with gamete maturation fertilization and preimplantation embryo development (Table 4). Although, a subset of these proteins identified are already supported by functional evidence in the literature from different species, we identified several proteins and associated physiological pathways that have not been previously reported for this microenvironment. In sections below, we focus in brief on the relevance of these results and discuss their importance in understanding fertility in cattle.

**Table 1:** Growth factors and cytokines in the oviductal cell secretions.

Protein name	Accession	OF	OEC-48	OEC-S4
***Growth factors***				
Bone morphogenetic protein 7*	F1MLT0		●	
Connective tissue growth factor	O18739		●	●
C type lectin domain family 11, member A	A5D7L1		●	
Endothelial cell specific molecule 1	A5D7V3	●	●	●
Fibroblast growth factor 18	Q0VCA0		●	
Fibroblast growth factor 21*	E1BDA6		●	
Glia maturation factor beta	P60984	●	●	●
Granulin	E1BHY6		●	●
Growth arrest specific 6	F1MZ40	●	●	●
Growth differentiation factor 15	E1BBL5		●	
Hepatocyte growth factor	Q76BS1			●
Hepatoma derived growth factor	Q9XSK7	●	●	●
Inhibin, beta A chain	P07995		●	
Kit ligand	Q28132		●	
Macrophage colony stimulating factor 1	F1MGS9		●	●
Midkine^a^	Q9N0E6		●	
Nephroblastoma overexpressed	Q2HJ34		●	
Nicotinamide phosphoribosyltransferase*	F1MJ80			●
Pigment epithelium derived factor	Q95121	●		
Platelet derived growth factor C	E1BJY4	●	●	
Transforming growth factor beta 1	P18341	●	●	
Transforming growth factor beta 2	P21214		●	
Vascular endothelial growth factor A	P15691		●	●
***Cytokines***				
Complement component 5a	F1MY85	●		
C-X-C motif chemokine 16	Q29RT9		●	●
C-X-C motif chemokine 6	P80221		●	
Dickkopf wnt signaling pathway inhibitor 3	A6QL81		●	●
Family with sequence similarity 3, member B	E1BQ21	●	●	
Family with sequence similarity 3, member C	A5PKI3		●	●
Family with sequence similarity 3, member D	E1BDN9		●	
Growth regulated protein homolog gamma	O46675		●	
Interleukin 8	P79255		●	
Leukemia inhibitory factor	Q27956		●	●
Macrophage migration inhibitory factor	P80177	●	●	
Myeloid derived growth factor	P62248		●	●
Osteopontin	P31096		●	●
Small inducible cytokine subfamily E, member 1	Q3ZBX5	●	●	●
Tumor necrosis factor ligand 1B	E1BF06		●	

* Exceptions: Proteins identified with only one unique peptide

**Table 2:** Proteases and protease inhibitors in the oviductal cell secretions.

Protein name	Accession	OF	OEC-48	OEC-S4
***Proteases***				
Cathepsin A	Q3MI05		●	●
Cathepsin B	P07688	●	●	●
Cathepsin C	F1N455		●	●
Cathepsin D	F1MMR6		●	●
Cathepsin V	P25975	●	●	●
Cathepsin Z	P05689		●	●
Coagulation factor II	P00735	●		●
Complement C1S	Q0VCX1	●		
Complement C2	Q0V7N2	●	●	
Complement C3	Q2UVX4	●	●	●
Complement factor B	P81187	●	●	●
Complement factor D	Q3T0A3	●	●	
Furin	Q28193		●	
Gamma glutamyl hydrolase	A7YWG4		●	
Granzyme A	F6QZF5		●	
Haptoglobin	Q2TBU0	●		
Serine protease HTRA 1	F1N152	●	●	●
Kallikrein related peptidase 10	Q0VCZ4		●	●
Lactotransferrin	P24627	●	●	●
Legumain	Q95M12		●	
Matrix metalloproteinase 1	F1MT97		●	
Matrix metalloproteinase 2	Q9GLE5		●	
Plasminogen	E1B726	●	●	●
Protein disulfide isomerase	A5D7E8	●	●	●
Tissue type plasminogen activator	Q28198	●	●	●
Tripeptidyl peptidase I	Q0V8B6		●	
Urokinase type plasminogen activator	Q05589		●	●
***Protease Inhibitors***				
Alpha-1-antiproteinase	P34955	●	●	●
Alpha-2-antiplasmin	P28800	●		●
Alpha-2-HS-glycoprotein	P12763	●	●	●
Alpha-2-macroglobulin	Q7SIH1	●	●	●
Angiotensinogen	Q3SZH5	●		
Antithrombin III	P41361	●	●	●
Cystatin B	F6QEL0	●	●	●
Cystatin C	P01035	●	●	●
Metalloproteinase inhibitor 1	P20414	●	●	●
Metalloproteinase inhibitor 2	F1N430	●	●	●
Metalloproteinase inhibitor 3	P79121		●	●
Serpin A3-1	Q9TTE1	●		
Serpin A3-7	A2I7N3	●	●	●
Serpin A3-8	A6QPQ2	●		
Serpin D1	A6QPP2	●		
Serpin H1	Q2KJH6	●	●	●

**Table 3:** Other enzymes identified in the oviductal cell secretions.

Protein name	Accession	OF	OEC-48	OEC-S4
Acid ceramidase	Q17QB3		●	●
Alpha amylase	F1MJQ3	●		
Alpha lactalbumin	P00711			●
Alpha-N-acetyl-galactosaminidase	Q1RMM9		●	●
Alpha-N-acetyl-glucosaminidase	A6QM01		●	
Angiogenin 1	P10152		●	
Apolipoprotein A-1 binding protein	Q6QRN6	●		●
Arylsulfatase A	Q08DD1	●	●	
Arylsulfatase B	A6QLZ3		●	●
Beta galactosidase	Q58D55		●	
Beta hexosaminidase	H7BWW2		●	●
Beta hexosaminidase subunit alpha	Q0V8R6		●	●
Beta mannosidase	Q29444		●	
Biotinidase	A6QQ07		●	●
Carbonic anhydrase IV	Q95323		●	●
Chitinase 3 like protein 1	G3X7D2	●	●	
Coagulation factor V	Q28107	●	●	●
Egf containing fibulin like extracellular matrix protein 1	A2VE41	●	●	●
Egf domain-specific O-linked N-acetyl-glucosamine transferase	A0JND3			●
Endoplasmic reticulum protein 44	Q3T0L2	●	●	●
Ero1 like protein	A5PJN2		●	●
Gamma interferon inducible lysosomal thiol reductase	F1MAU3		●	●
Glucose regulated protein 78	Q0VCX2	●	●	●
Glucosidase 2 subunit beta	Q28034	●	●	●
Glutaminyl peptide cyclotransferase	Q28120		●	
Heparanase	F1N1G1		●	
Interferon, gamma inducible protein 30	A6QPN6		●	●
Lysyl oxidase homolog 4	Q8MJ24		●	●
Peptidylprolyl isomerase C	Q08E11		●	●
Peroxiredoxin 4	Q9BGI2	●	●	●
Phospholipid transfer protein	Q58DL9	●		
Procollagen lysine, 2-oxoglutarate 5-dioxygenase 1	O77588		●	●
Procollagen lysine, 2-oxoglutarate 5-dioxygenase 2	A7MB83		●	●
Prolyl 4-hydroxylase subunit alpha 1	A6QL77			●
Prostaglandin-H2 D-isomerase	O02853	●	●	
Protein disulfide isomerase	A6H7J6	●	●	●
Protein disulfide isomerase A4	F1MEN8	●	●	●
Protein O-fucosyltransferase 1	Q7YRE7			●
Protein O-glucosyltransferase 1	Q5E9Q1			●
Ribonuclease 4	Q58DP6	●	●	
Serpin peptidase inhibitor, clade C, member 1	P41361	●	●	●
Stromal cell derived factor 2	Q3SZ45			●
Sulfhydryl oxidase	F1MM32	●	●	●
Superoxide dismutase	A3KLR9	●	●	●
Tissue alpha-L-fucosidase	Q2KIM0		●	
Vanin 1	Q58CQ9	●	●	

**Table 4:** Other proteins associated with gamete maturation, fertilization and preimplantation embryonic development in the oviductal cell secretions.

Protein name	Accession	OF	OEC-48	OEC-S4
Alpha-1-acid glycoprotein	Q3SZR3	●		●
Apolipoprotein A-I	P15497	●	●	●
Apolipoprotein A-IV	F1N3Q7	●		
Apolipoprotein C-III	P19035	●		
Apolipoprotein D	Q32KY0	●		
Apolipoprotein H	P17690	●		●
Calreticulin	P52193	●	●	●
Endoplasmin	Q95M18	●	●	●
Fetuin B	Q58D62	●		●
Fibronectin	P07589	●	●	●
Oviduct specific glycoprotein	Q28042	●	●	●
Prosaposin	P26779	●	●	●
Serum Albumin	P02769	●	●	●
Zinc alpha-2-glycoprotein	Q3ZCH5	●	●	●
Complement C5a	F1MY85	●		●
Follistatin	P50291		●	●
Gelsolin	F1N1I6	●	●	●

### Growth factors and cytokines

Developmental functions that occur in the oviductal microenvironment are known to be supported by factors that signal to gametes or embryo in the lumen. Among the proteins classified as growth factors and cytokines (Table 1), only 15 have been linked in previous reports to potential functions in the oviduct. The remaining 24 proteins were candidates detected in this microenviroment for the first time, the functional significance of which remains to be uncovered. Expression of macrophage colony stimulating factor 1 (CSF1) has been reported to increase after lipopolysaccharide exposure in the bovine oviductal epithelium [47]. CSF1 has been shown to accelerate development in bovine embryos [48, 49]. Although function remains unclear, hepatocyte growth factor has been reported in human oviductal fluid [50]. Activin/inhibin subunits have been identified in the murine oviduct as responsible for stimulating early embryogenesis [51]. During bovine embryogenesis, platelet derived growth factor (PDGF) is known to stimulate development during the fourth cell cycle [52]. Transforming growth factor β and vascular endothelial growth factor have been previously identified in the bovine oviduct [53], and may play a role in the developmental competence of bovine oocytes [54], and promote early embryonic development [55]. It has been suggested that BMP signaling is involved in crosstalk between the oviduct and the embryo during early stages of development [56].

Cytokines that included several inflammation-associated candidates were found to be expressed by oviductal cells (Table 1). Only few of these have been previously reported in the oviduct for any species. Early studies have demonstrated synthesis of leukemia inhibitory factor (LIF) by bovine oviductal cells [57]. LIF expression is known to have beneficial effects on sheep oocytes [58], and early embryos [59]; similar results have been recently reported for cattle [49, 60]. Interleukin 8 expression, often connected to inflammation, has been reported in human fallopian tubes [61]. Macrophage migration inhibitory factor has been identified in bovine oviducts with higher levels detected in the postovulatory phase, but its function remains unclear [62]. Previous studies have detected and linked osteopontin expression in the bovine oviduct [63], with a role in sperm-egg binding and fertilization [64]. Conserved functions for osteopontin have also been reported in porcine [65], and murine oviducts [66]. The tumor necrosis factor α system has been suggested to be responsible for local contractions modulating transport of the gametes and embryos [67].

### Proteases and protease inhibitors

Protease activity has been reported in oviducts in several species, and some of their functions have been linked to sperm capacitation [68, 69]. We identified 27 proteases and 16 protease inhibitors expressed by oviductal cells (Table 2). Cathepsins are considered to be involved in gamete maturation leading to fertilization, and we identified several cathepsins (A, B, C, D, V and Z) produced by the oviductal cells. Previous studies have reported cathepsins in the oviducts of domestic cats [70], hamsters [71], and llamas [72]. Components of the complement pathway were also identified (S1 Fig). Complements have been suggested to be important for sperm-oocyte interaction [73, 74]; complement components are also known to be activated by spermatozoa and could cause acrosome loss in rabbit spermatozoa [75]. Complements have also been demonstrated to stimulate embryo development [76, 77]. Although furin has not been reported in the oviductal secretions, its role in protein processing in the epididymal fluid has been suggested [78]. Haptoglobin mRNA has been previously reported in the oviduct of cycling cows [79]. Kallikrien-related peptidases have been linked to a role in host defence in cervical mucus [80]. Lactotransferrin has been reported in human oviducts as a modulator of gamete interaction [81].

The proteases plasminogen, tissue type plasminogen activator, urokinase type plasminogen activator, and the protease inhibitor α-2-antiplasmin were identified in the oviductal secretions. Plasminogen/plasmin system has been suggested to regulate sperm entry into the oocyte in multiple species [82, 83]. In addition, α2 macroglobulin known to inhibit proteases from all catalytic classes was also identified in the oviductal cell secretions for the first time. An immunoprotective role for placenta-sourced α2 macroglobulin has been suggested for this acute phase protein in rats [84]. Other candidates that regulate extracellular matrix remodeling were also present in the oviductal secretions (S2 Fig). Matrix metalloproteinases (MMP 1 and 2), and corresponding tissue inhibitors of metalloproteinases (TIMP 1, 2 and 3) were identified in the oviductal secretome. Differential expression for MMPs and TIMPs have been reported in the bovine oviduct during the estrous cycle [85], with potential effects across fertilization and early embryonic development.

Inhibitors of acrosomal and other lysosomal proteases like α-1-antiproteinase/antitrypsin and different serpins (A3-1, A3-7, A3-8, D1 and H1), cystatins (B and C) were identified. Similar inhibitors of acrosomal proteases has been reported in oviductal fluid collected from the rhesus monkey [86] and the rabbit [87].

### Other enzymes

In addition to the proteases indicated above, the oviductal cell secretions contained numerous enzymes (Table 3). Of these, only a few have been previously identified in the oviduct in different species. Ceramide metabolism mediated by acid ceramidase has been demonstrated to be critical for early embryo survival in mice [88]. Glycosidase activities in bovine oviductal fluid have been reported in previous studies [89]. These enzymes have been associated with modifications to the oocyte zona pellucida and capacitation of spermatozoa. In addition to glucosidase 2 reported in sheep [90] and cows [23], this study also identified 6 specific enzymes that may be linked to specific carbohydrate modifications previously measured in the oviductal fluid [89]. Arylsulfatases (A and B) have been reported in the rabbit oviduct [91], and indicate potential for glycoconjugate formation in this microenvironment [92]. Carbonic anhydrase IV has been demonstrated to provide an essential role in bicarbonate mediated activation of human and murine sperm [93, 94]. Chitinase-like proteins have been previously reported in the sheep oviduct [95]. Chitinase 3 like protein 1 has been reported to regulate inflammation and tissue remodeling [96]. Glucose-regulated protein 78 secreted in the human oviduct has been demonstrated to decrease sperm zona pellucida binding [97]. Heparan sulfate proteoglycans and their binding proteins have been found to be important in the bovine reproductive physiology. Heparanase has not been previously reported in the oviduct, but its function in the uterus during implantation has been well studied in murine models [98, 99]. Phospholipid transfer protein expression is known to be stimulated in response to embryos in the murine oviduct [100]. Superoxide dismutase expression was also detected in the oviductal cell secretions indicating antioxidant defense by reducing superoxide radicals in this microenvironment. Superoxide dismutases have been previously reported in the bovine oviduct [101], and its importance in redox regulation has been emphasized in several species.

### Other proteins associated with gamete maturation, fertilization and preimplantation embryo development

Among proteins that did not fall into one of the above categories, we identified several that are of functional importance for gamete regulation, fertilization and preimplantation embryonic development (Table 4). In male fertility, apolipoprotein A-1 has been associated with sperm motility [102]. Apolipoproteins can act as cholesterol acceptors that facilitate cholesterol efflux from plasma membrane of spermatozoa [103], a necessary event for capacitation/hyperactivation. Specific association of apolipoprotein A-1 to bovine seminal plasma proteins [104] and modulation of sperm capacitation [105] have been previously demonstrated. Expression of apolipoproteins in the oviduct has been reported in different mammalian model systems: Apolipoprotein D has been detected in guineapig oviducts [106]. Apolipoprotein A-1 has been detected in rat oviducts [107]. An apolipoprotein H-like protein has been purified from human follicular fluid [108]. In addition to apolipoproteins A-1 and D, apolipoproteins A-IV, C-III, and H that were detected in this study have not been previously reported in mammalian oviducts.

The oviductal secretome also contained factors that have been associated with sperm survival, transport, and signal transduction during fertilization. α-1-acid glycoprotein has been reported to inhibit neutrophil phagocytosis of sperm in the bovine oviduct [109]. Fibronectin has been demonstrated to stimulate human sperm capacitation by activating the protein kinase A pathway [110]. Organization of the extracellular matrix and paracrine communication by fibronectin has been identified to be important for early embryogenesis [111, 112]. Zinc-α-2-glycoprotein has been demonstrated to induce cAMP signaling and modulate motility in human sperm [113]. Calreticulin has been suggested to interact with the murine oocyte and mediate signaling linked to cell cycle resumption [114]. Phosphorylation of endoplasmin bound to murine spermatozoa has been associated zona pellucida interactions preceding fertilization [115]. Fetuin B is vital for maintaining fertility of murine ovulated oocytes by blocking ovastacin, a cortical granule protease known to trigger zona pellucida hardening [116]. Albumin has been found to increase blastocyst development in individual culture of bovine embryos [117]. Follistatin has been shown to be important for bovine early embryo development [118, 119]. Complement C5a has been shown to support human embryonic stem cell pluripotency even in the absence of other growth factors [120] (S1 Fig).

## Conclusions

The oviduct presents a crucial site for gamete regulation including sperm capacitation, oocyte maturation, fertilization and preimplantation development of the early embryo. This study represents a comprehensive documentation of the bovine oviductal secretions comparing both *ex vivo* intact oviducts and *in vitro* oviductal epithelial cells. It is of interest to note that transudation from plasma contributes little to the defining characteristics of this luminal microenvironment in cattle. The secreted protein profile established for the oviductal fluid in this manuscript forms the foundation for future functional studies for both advancing basic understanding and making improvements to reproduction technologies in cattle. Our proteomics database will also serve as a long-term reference for addressing a variety of questions regarding the bovine oviduct, and seed new discoveries and linkages over time; perhaps aspects that we failed to appreciate given the current state of understanding will manifest with parallel advancements to bovine reproductive function. Some areas not distinguished by this study are the changes that may occur to the oviductal microenvironment with the estrous cycle [16, 121] and microvesicles/exosomes present in the oviductal fluid that may deliver proteins to sperm and regulate its functional activation [122, 123]. These remain important topics for future investigations towards refining understanding of bovine oviductal physiology.

## Supporting information

S1 Fig Model for regulation of extracellular matrix remodeling in the oviductal microenvironment. (PDF)

S2 Fig Model for regulation of complement system that may enhance embryo development in the oviductal microenvironment. (PDF)

S1 Table The 68 secreted proteins in common between OF, OEC-48 and OEC-S4. (PDF)

S2 Table Secreted proteins identified in OF, OEC-48 and OEC-S4 whose corresponding transcripts were not detected in the oviduct. (PDF)

S1 Dataset Database of total proteins and total secreted proteins from OF, OEC-48 and OEC-S4. (XLSX)

S1 Movie Extruded epithelial layer from the bovine oviduct containing ciliated cells. (MP4)
