# Supplementary material for: Profiling of proteins secreted in the bovine oviduct reveals diverse functions of this luminal microenvironment
Source: PLoS One. 2017 Nov 20;12(11):e0188105. doi: 10.1371/journal.pone.0188105 (PMC5695823; doi:10.1371/journal.pone.0188105)
Supplement: S1 Fig — (PDF) [file pone.0188105.s001.pdf]

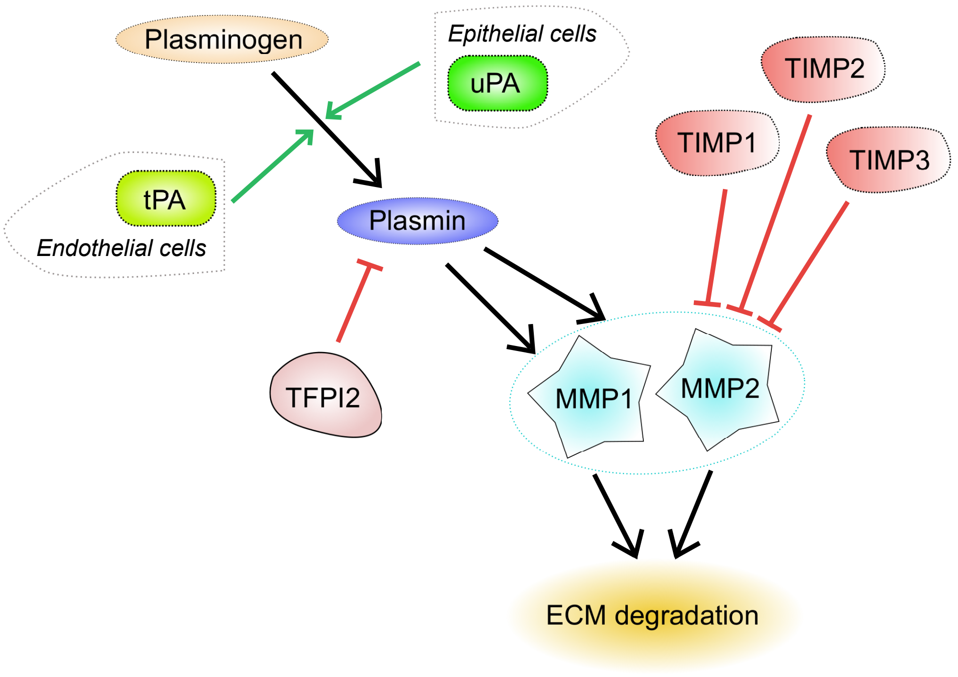

Figure S1. Model for regulation of extracellular matrix remodeling in the oviductal microenvironment. Proteins identified in the bovine oviductal secretions involved in pathways controlling extracellular matrix remodeling are indicated. Plasminogen is converted to plasmin through the activities of urokinase-type plasminogen activator (uPA) and the tissue-type plasminogen activator (tPA). Plasmin activates pro-matrix metalloproteinases (MMPs), and may target MMP1 and MMP2 in this microenvironment. MMPs play an important role in degradation of the extracellular matrix (ECM). Plasmin activity could be inhibited by tissue factor pathway inhibitor 2 (TFPI2), and different serine peptidase inhibitors (SERPINs) identified. Tissue inhibitors of metalloproteinases (TIMP1, TIMP2 and TIMP3) negatively regulate MMP and fine tune ECM modifications. In synergy, the anti-proteinase $\alpha 2$ macroglobulin (A2M) could also bind and inhibit active MMPs.
