# Supplementary material for: Profiling of proteins secreted in the bovine oviduct reveals diverse functions of this luminal microenvironment
Source: PLoS One. 2017 Nov 20;12(11):e0188105. doi: 10.1371/journal.pone.0188105 (PMC5695823; doi:10.1371/journal.pone.0188105)
Supplement: S2 Fig — (PDF) [file pone.0188105.s002.pdf]

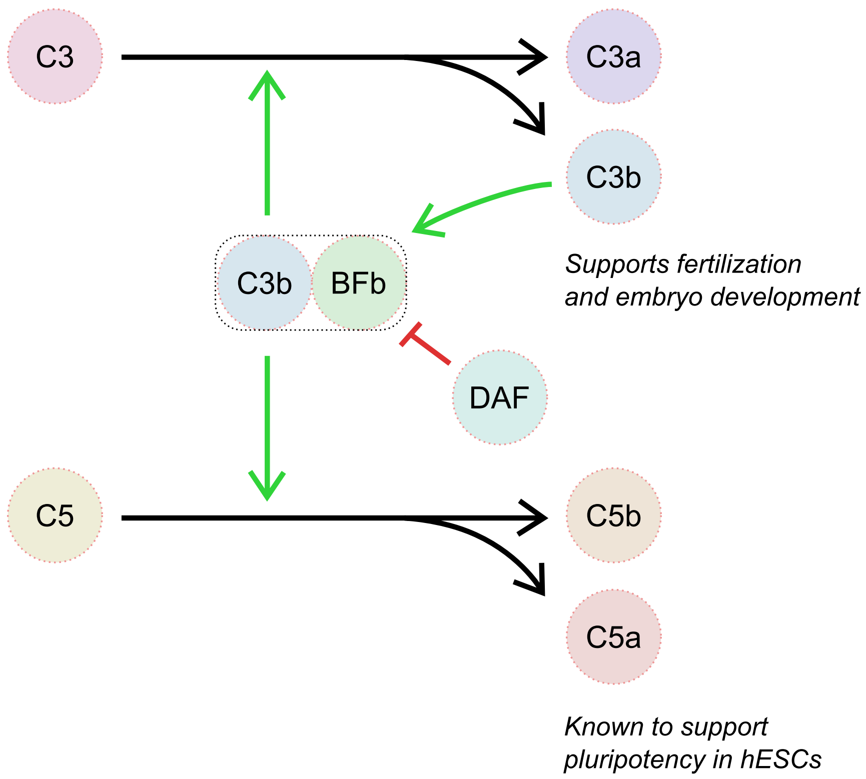

Figure S2. Model for regulation of complement system that may enhance embryo development in the oviductal microenvironment. Several members of the complement system were identified in the bovine oviductal secretion. Distinct from the classical pathway in innate immunity, complements can be interpreted to have alternate functions in the bovine oviduct. Generation of C3b could be linked to fertilization and stimulating preimplantation embryo development. Generation of C5a could be linked to pluripotency sustenance as observed in studies on human embryonic stem cells.
