# Supplementary material for: Profiling of proteins secreted in the bovine oviduct reveals diverse functions of this luminal microenvironment
Source: PLoS One. 2017 Nov 20;12(11):e0188105. doi: 10.1371/journal.pone.0188105 (PMC5695823; doi:10.1371/journal.pone.0188105)
Supplement: S1 Table — (PDF) [file pone.0188105.s003.pdf]

Table S1. The 68 secreted proteins in common between OF, OEC-48 and OEC-S4

A2M, AHSG, ALB, APOA1, AZGP1, B2M, C3, CALR, CALR, CD55, CFB, CLEC3B, CLU, COL12A1, CST2, CST3, CTSB, CTSL, CUTA, DNAJC3, ECSOD, EFEMP1, ERP44, ESM1, F5, FN1, GAS6, GC, GPC1, GSN, HDGF, HSP90B1, HSPA5, HTRA1, IGFBP2, IGFBP6, IL1RAP, LTF, MANF, MFAP5, MFGE8, MSLN, NPC2, NUCB1, OVGP1, P4HB, PDIA3, PDIA4, PLAT, PLG, PLG, PRDX4, PRKCSH, PSAP, QSOX1, RARRES1, RBP4, SCYE1, SDF4, SERPINA1, SERPINA3-7, SERPINC1, SERPINC1, SERPINH1, SPARC, TFPI2, TIMP1, TIMP2
