# Supplementary material for: Profiling of proteins secreted in the bovine oviduct reveals diverse functions of this luminal microenvironment
Source: PLoS One. 2017 Nov 20;12(11):e0188105. doi: 10.1371/journal.pone.0188105 (PMC5695823; doi:10.1371/journal.pone.0188105)
Supplement: S2 Table — (PDF) [file pone.0188105.s004.pdf]

Table S2. Secreted proteins identified in OF, OEC-48 and OEC-S4 whose corresponding transcripts* were not detected in the oviduct

Name	Accession
Alpha amylase	F1MJQ3
Beta hexosaminidase	H7BWW2
Cathepsin B	P07688
Cathepsin D	F1MMR6
Cystatin B	F6QEL0
Folate receptor alpha	P02702
Glucosidase 2 subunit beta	Q28034
Granzyme A	F6QZF5
Growth regulated protein homolog gamma	O46675
Hepatoma derived growth factor	Q9XSK7
Interleukin 8	P79255
Interstitial collagenase	F1MT97
Kininogen 2	P01045
Lysozyme C, milk isozyme	Q6B411
Macrophage migration inhibitory factor	P80177
Myeloid derived growth factor	P62248
NAD(P)H-hydrate epimerase	Q6QRN6
Paraoxonase 1	Q2KIW1
Peptidyl-prolyl cis-trans isomerase C	Q08E11
Platelet derived growth factor C	E1BJY4
Primary amine oxidase, liver isozyme	Q29437
Protein disulfide-isomerase A4	F1MEN8
Protein HP-20 homolog	Q2KIT0
Protein HP-25 homolog 1	Q2KIX7
Serpin A3-7	A2I7N3
Serpin A3-8	A6QPQ2
Small inducible cytokine subfamily E member 1	Q3ZBX5
Tumor necrosis factor ligand 1B	E1BF06
Family with sequence similarity 3, member B	E1BQ21
Family with sequence similarity 3, member D	E1BDN9

* Transcriptome datasets are from Maillo *et al.* [32] and Gonella-Diaza *et al.* [33]
